# Automated Quantitative Stress Perfusion Cardiac Magnetic Resonance in Pediatric Patients

**DOI:** 10.3389/fped.2021.699497

**Published:** 2021-09-03

**Authors:** Cian M. Scannell, Hadeer Hasaneen, Gerald Greil, Tarique Hussain, Reza Razavi, Jack Lee, Kuberan Pushparajah, Phuoc Duong, Amedeo Chiribiri

**Affiliations:** ^1^School of Biomedical Engineering and Imaging Sciences, St Thomas' Hospital, King's College London, London, United Kingdom; ^2^Department of Pediatrics, University of Texas Southwestern Medical Center, Dallas, TX, United States

**Keywords:** cardiac magnetic resonance, automated quantitative stress perfusion, deep learning, pediatrics, Kawasaki disease

## Abstract

**Background:** Myocardial ischemia occurs in pediatrics, as a result of both congenital and acquired heart diseases, and can lead to further adverse cardiac events if untreated. The aim of this work is to assess the feasibility of fully automated, high resolution, quantitative stress myocardial perfusion cardiac magnetic resonance (CMR) in a cohort of pediatric patients and to evaluate its agreement with the coronary anatomical status of the patients.

**Methods:** Fourteen pediatric patients, with 16 scans, who underwent dual-bolus stress perfusion CMR were retrospectively analyzed. All patients also had anatomical coronary assessment with either CMR, CT, or X-ray angiography. The perfusion CMR images were automatically processed and quantified using an analysis pipeline previously developed in adults.

**Results:** Automated perfusion quantification was successful in 15/16 cases. The coronary perfusion territories supplied by vessels affected by a medium/large aneurysm or stenosis (according to the AHA guidelines), induced by Kawasaki disease, an anomalous origin, or interarterial course had significantly reduced myocardial blood flow (MBF) (median (interquartile range), 1.26 (1.05, 1.67) ml/min/g) as compared to territories supplied by unaffected coronaries [2.57 (2.02, 2.69) ml/min/g, *p* < 0.001] and territories supplied by vessels with a small aneurysm [2.52 (2.45, 2.83) ml/min/g, *p* = 0.002].

**Conclusion:** Automatic CMR-derived MBF quantification is feasible in pediatric patients, and the technology could be potentially used for objective non-invasive assessment of ischemia in children with congenital and acquired heart diseases.

## Introduction

Cardiac magnetic resonance (CMR) has been gaining in importance as a technique for the assessment of a wide variety of congenital and acquired heart diseases in children. It provides both functional and anatomical assessment, with good spatial resolution, without exposing patients to ionizing radiation. This is of particular importance as children are more likely to develop radiation-induced cancer than adults (1) and they frequently require multiple follow-up scans to monitor disease progression or the effects of an intervention. CMR has been applied to the assessment of the great vessels using contrast-enhanced CMR angiography (CMRA) (2–5) and recent technical advances have made non-contrast CMRA possible (6, 7). CMRA is now widely used for the diagnosis of vascular diseases in pediatrics, frequently in place of an invasive coronary catheterization (1, 8). CMR has been further used for cardiac chamber quantification (9), flow quantification (10), and the assessment of myocardial perfusion (11–15).

Stress perfusion CMR has been repeatedly shown to possess both good sensitivity and good specificity for the non-invasive assessment of myocardial ischemia (16–19). Although it is known that myocardial ischemia can occur in pediatric patients, the aforementioned studies have been conducted in adult populations and there is still limited data on the efficacy of the technique in pediatric populations (11). The feasibility of stress perfusion CMR in pediatrics has been long since reported (11, 20–24) and retrospective studies have reported a high diagnostic accuracy for the detection of ischemia (13, 25). The limitation of work in this field is that it has been primarily conducted in highly specialized centers with significant local expertise, and this high diagnostic accuracy may not translate to less specialized centers.

The quantification of myocardial blood flow (MBF) from stress perfusion CMR is possible and has been shown to be of high diagnostic and prognostic value (26, 27). Recently, methods for the automated analysis of the images have become available (28, 29), including robust motion compensation (30), reducing the need for expert operators with high levels of training. As such, the fully automated quantification of MBF may facilitate the adoption of stress perfusion CMR in clinical routine. In this study, we aimed to assess the feasibility of automated high-resolution quantitative stress perfusion CMR in pediatric patients.

## Methods

### Study Population

Pediatric cardiac MRI perfusion datasets from a single institution (Evelina London Children's Hospital, United Kingdom), acquired between 2010 and 2018 were retrospectively analyzed. This study was performed under the ethical approval of the local ethics committee (Ethics No. RJ109/N112). Patients who underwent dual-bolus perfusion protocol (31) were included in this study. These patients had congenital heart disease affecting the coronary arteries (anomalous origin of the left coronary artery arising from the pulmonary artery (ALCAPA), malignant course of left coronary artery arising from the inappropriate sinus, repaired tetralogy of Fallot with known coronary artery proximity with main pulmonary artery) or acquired heart disease such as Kawasaki disease. Due to the available expertise in pediatric CMR in this institution, coronary artery anatomy was predominantly imaged using CMR as reference standard. Computed tomography (CT) angiogram or invasive fluoroscopic angiogram were carried out if further correlation was needed or if the MR image quality was deemed inadequate.

Coronary artery aneurysms are assessed according to the American Heart Association scientific statement 2017 (32), with a z-score cut off point of ≥2.5 to define abnormality, based on the anatomical images by an experienced operator (PD). Aneurysms were classified on the basis of absolute dimensions. Dilation or small aneurysms are defined as a localized dilation of the internal lumen diameter but < 4 mm. Medium aneurysms are defined as an internal lumen diameter ≥4 mm but ≤8 mm. Large or giant aneurysms are defined as those with an internal lumen diameter >8 mm.

All patients had anatomic coronary artery changes (stenosis or dilatation) and either symptoms of angina or electrocardiographic evidence of ischemic changes at rest or during exercise.

### CMR Perfusion Imaging

Perfusion images were acquired in three left ventricular (LV) short-axis slices (apical, mid-cavity, and basal) at mid-expiration when possible. Due to the young age of the patients and the potential side effect of adenosine infusion, younger patients required general anesthesia during CMR study, with volatile anesthetics (isoflurane and sevoflurane). Heart rate lowering medications, such as remifentanil, were not used. Where the heart rate did not permit the acquisition of three slices in every heartbeat, the basal and mid-cavity slices were acquired. Imaging was performed with a saturation-recovery acquisition sequence on either a 1.5T (Ingenia, Philips Healthcare, Best, The Netherlands) or 3T system (Achieva, Philips Healthcare, Best, The Netherlands). The typical imaging parameters were at 1.5T with a 12-channel cardiac phased array receiver coil: balanced gradient echo readout, repetition time/echo time 3.0 ms/1.5 ms, flip angle 50°, saturation-recovery delay 100 ms and at 3.0T with a 32-channel coil: gradient echo readout, repetition time/echo time 2.5 ms/0.9 ms, flip angle 20°, saturation-recovery delay 120 ms. Five-fold *k-*t sensitivity encoding (*k-t* SENSE) acceleration with 11 training profiles was used to achieve a representative spatial resolution of 1.25 × 1.25 × 10 mm^3^. Stress images were acquired during adenosine-induced hyperemia (140 μg/kg/min). 0.075 mmol/kg of bodyweight gadolinium (Gd) extracellular contrast agent (gadobutrol, Gadovist, Bayer, Germany) was injected at 4 mL/s followed by a 20-mL saline flush for each perfusion acquisition. Each bolus of gadobutrol was preceded by a diluted pre-bolus with 10% of the dose to allow quantification of perfusion, according to published methods (31).

### Image Processing

The perfusion images were corrected for respiratory motion (30) and processed fully-automatically using our deep learning-based processing pipeline (29). In light of the smaller heart sizes, more conservative myocardial segmentations are needed, as compared to in adults. For this reason, test-time data augmentation was employed. That is that, where multiple segmentations are computed for different transformed versions of the image and a pixel is deemed to be in the myocardium if it is predicted to be in the myocardium 8/10 times. Pixel-wise time signal intensity curves were extracted from the myocardial mask and signal intensity curves were subsequently split into the time intervals corresponding to the pre bolus injection and the main bolus injection for quantification. Quantitative MBF was estimated on a pixel-wise level by fitting the observed arterial input function and myocardial tissue curves to a two-compartment exchange model (33), as commonly used for quantitative myocardial perfusion analysis (34). For the relation of pixel-wise MBF estimates to coronary artery territories, pixels were assigned to standard American Heart Association (AHA) segments using the automatically computed right ventricular insertion points and AHA segments were assigned to their respective perfusion territory (35). The MBF value recorded for a specific coronary perfusion territory was the mean value of the 2 lowest segments in that territory, as previously validated (26).

### Statistical Analysis

The descriptive statistics are presented as mean ± standard deviation (SD) or median [inter-quartile range (IQR)] for non-normally distributed variables. The distributions of quantitative MBF values are compared using the non-parametric Mann-Whitney U test. The significance level α = 0.05 was used to determine statistical significance. This analysis was performed using SciPy (36).

## Results

A total of 42 patients underwent stress perfusion cardiovascular magnetic resonance scans between 2010 and 2018. Fourteen patients (with 16 scans) underwent scans being performed with a dual-bolus acquisition and thus were included in the study. Fourteen scans were done at 1.5 T and 2 scans were done at 3 T. Two patients underwent a repeat stress perfusion scan for follow up. 12/16 scans were performed under general anesthesia. The patient's demographic and baseline data are given in Table 1. In 3/16 cases images were acquired in two rather than three LV slices as high heart rates prevented the acquisition of three slices in a single R-R interval. Four scans were visually positive for perfusion defects (in a total of 14 AHA segments) with a perfusion abnormality identified in the left anterior descending (LAD) territory in 2 cases and in the left circumflex (LCx) territory in the other 2 cases. MBF for 15/16 stress perfusion CMR scans were assessed quantitatively, one was excluded due to the presence of severe parallel imaging artifacts in the reconstructed images The automated image processing was successful in all these 15 cases. Rest images were not analyzed as they were not acquired in all cases.

**Table 1:** Demographic and baseline data.

	***N* = 16 examinations/*N* = 14 patients**
Age (years) [median (IQR)]	8.0 (3.5, 13.75)
Gender	7 male (50%)
Weight (kg) [median (IQR)]	28.45 (17.2, 58.75)
BMI (kg/m^2^) [median (IQR)]	18.8 (16.1, 22.1)
**Systolic function**	
Normal (LVEF > 55%)	13 (81.3%)
Impaired	3 (18.8%)
LVEDI (ml/m^2^) (mean ± SD)	76.3 ± 25.8
LVEF (%) (mean ± SD)	58.4 ± 8.9
**Diagnosis**	
Kawasaki Disease	11 (68.8%)
Reimplantation of Left Coronary Artery (LCA) (ALCAPA), malignant LCA course	3 (18.8%)
Tetralogy of Fallot with abnormal LAD	1 (6.3%)
Neonatal myocardial infarction	1 (6.3%)
**Heart rate (beats per minute)**	
Rest (mean ± SD)	76.7 ± 14.7
Stress (mean ± SD)	99.8 ± 17.8

*Data are reported as N (percentage%), median [Interquartile range (IQR), or mean ± standard deviation (SD)]*.

**Figure 1:**
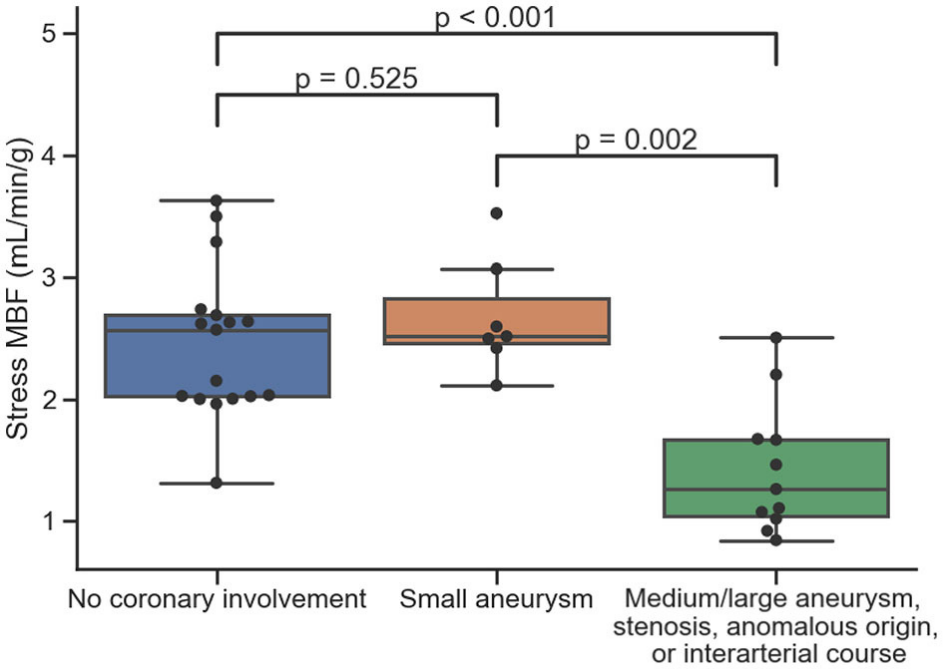
The distributions of quantitative MBF estimates on a per-coronary perfusion territory level for normal coronary vessels, small aneurysms, and medium/large aneurysms, stenosis, anomalous origin, or interarterial course, respectively.

**Figure 2:**
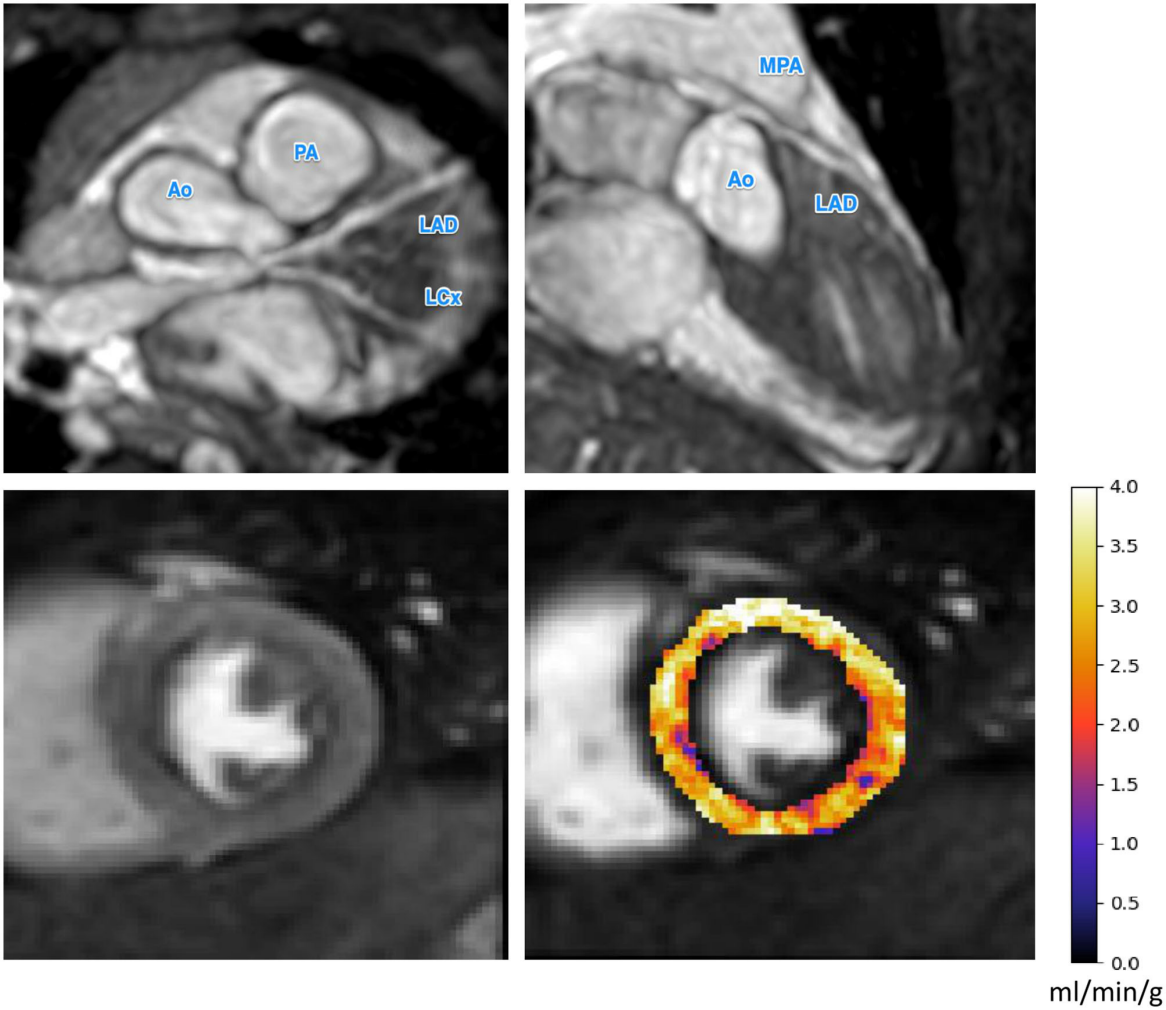
The CMR angiography (top row) and stress perfusion CMR for a patient with a small left main coronary artery aneurysm. The small aneurysm does not seem to inhibit perfusion as reflected by the uniformly high MBF values. Ao, Aorta; PA, pulmonary artery; MPA, main pulmonary artery; LAD, left anterior descending artery; LCx, left circumflex artery.

**Figure 3:**
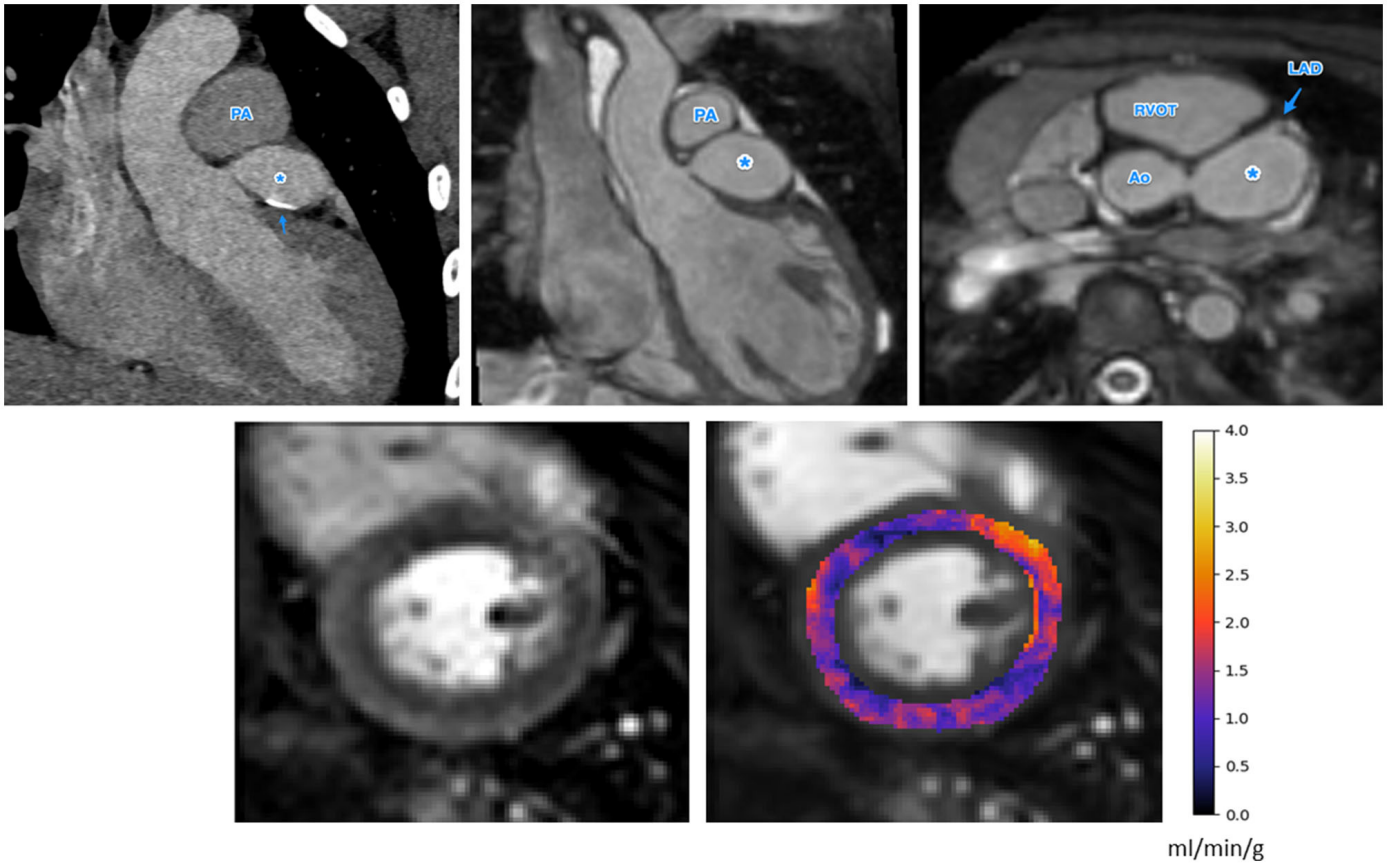
The anatomical images (top row, CT left, MR center and right) and stress perfusion CMR with quantitative MBF map (bottom row) for a patient with Kawasaki disease and a giant aneurysm of the LMCA, indicated as *in the anatomical images. There is also calcification of the LMCA seen on the CT image (top left), indicated by the arrow. There is globally low MBF values, possibly indicating microvascular dysfunction (bottom right). Ao, Aorta; PA, pulmonary artery; MPA, main pulmonary artery; RVOT, right ventricular outflow tract; LAD, left anterior descending artery.

The median pixel-wise MBF estimate across all subjects was 2.34 (1.82, 2.97) ml/min/g. The median pixel-wise MBF in patients with visual ischemia was 1.84 (1.34, 2.14) ml/min/g, which was significantly lower than that in patients with no visual perfusion defect 2.48 (1.97, 3.04) ml/min/g (*p* < 0.001). The MBF estimate in patients with Kawasaki disease but with no significant coronary involvement was 2.57 (2.02, 2.69) ml/min/g. This was not significantly different to the MBF [2.52 (2.45, 2.83) ml/min/g] in coronary territories with a small aneurysm *p* = 0.525. There was a reduction in MBF in coronary territories perfused by vessels with a medium/large aneurysm, a stenosis, an anomalous origin, or an interarterial course to 1.26 (1.05, 1.67) ml/min/g. This was significantly lower as compared to normal patients (*p* < 0.001) and territories with small aneurysm (*p* = 0.002) with the distributions of the per-coronary perfusion territory MBF values visualized in Figure 1. Figures 2, 3 show raw perfusion MR images with the pixel-wise MBF maps and corresponding anatomical images.

## Discussion

Myocardial ischemia in children is known to result from congenital coronary anomalies (20) as well as from acquired causes, including inflammatory diseases affecting the coronary arteries, such as Kawasaki disease (21). Untreated Kawasaki disease can lead to coronary artery aneurysms in ~25% of cases (32), predisposing to thrombosis, stenosis and occlusion (37). As such, the routine clinical adoption of myocardial perfusion imaging in the pediatric population has a huge potential benefit. It may allow the early identification of myocardial ischemia and help to avoid further adverse events, including irreversible myocardial dysfunction.

Stress perfusion imaging is potentially beneficial in these patients as it can assess the functional significance of partially occluded lesions and provide information on segments distal to the coronary artery aneurysms which may not appear occluded but are functionally different as compared to unaffected arteries. Alternative myocardial perfusion imaging modalities include single photon emission computed tomography (SPECT) and positron emission tomography (PET) but both subject the patient to ionizing radiation and are limited in spatial resolution as compared to CMR. Furthermore, adenosine stress perfusion CMR is considered to have a good safety profile amongst pediatrics (14), making it a strong candidate for clinical adoption.

The limitation of stress perfusion CMR is that it requires expertise to acquire and interpret the images (25, 38). This problem is likely to be exacerbated in pediatric populations due to lower image quality, inadequate spatial resolution to allow the transmural discrimination of perfusion defects, and higher susceptibility to imaging artifacts, such as dark rim, in the smaller myocardial wall. Despite the fact that all patients had anatomically affected coronary arteries, the visual assessment only reported perfusion defects in 4/16 scans. Based on the quantitative MBF values, this is likely an underestimation of the amount of ischemia present which highlights the difficulty of the interpretation and the need for the integration of quantitative perfusion CMR in clinical routine.

The results of our retrospective study show that fully automated quantification of first pass stress perfusion CMR is feasible in pediatrics and that the CMR measurements of stress MBF well match the corresponding anatomical images of the patients. For the example patient shown in Figure 3, with a giant LMCA aneurysm, global MBF is substantially reduced to 1.25 ml/min/g, as compared to the patient in Figure 2, who has a small LMCA aneurysm and global MBF of 2.98 ml/min/g. The widespread reduction in MBF seen in patients with large aneurysms may also lend support to previous findings of microvascular dysfunction in these patients (22, 25, 39, 40), as microvascular dysfunction is known to cause ischemia and reduced MBF by quantitative perfusion CMR (41). In particular, this may explain the extensive ischemia and the fact that it is not restricted to the perfusion territories of the affected coronary arteries. This theory is supported by previous findings of global reductions in myocardial perfusion that have been reported by visual (25) and semi-quantitative (22) stress perfusion CMR and PET MBF (39). The benefit of the quantitative perfusion analysis is also shown by the example in Figure 3 as it was reported visually as normal. Global MBF reductions are difficult to assess visually as there is little regional differences but are clear on the quantitative analysis. In contrast to the large aneurysms, mild dilatations or small aneurysms yielded MBF values comparable to cases with no macroscopic coronary artery involvement.

The technical difficulties to be dealt with in order to facilitate the widespread clinical adoption in pediatric patients include the need for higher temporal and spatial resolutions to cope with the higher heart rates and smaller heart size as compared to adults, patient motion and the inability to breath-hold. However, solutions to these challenges are becoming available. Accelerated MR image acquisitions and in particular, spatiotemporal data under-sampling approaches combined with multi-element coil spatial encoding have shown that it is feasible to acquire high resolution images at very high heart rates while reducing dark rim artifacts (42). Furthermore, advanced image analysis (29, 30) and robust quantification (33) methods have been developed which make the analysis fast, automated, and accurate. The image analysis pipeline required very little adjustment for its deployment in pediatrics despite the fact that it was developed in adults (29). This is likely because, despite the differences in cohorts, the acquisition protocols were very similar between the studies. Further work may be required to allow the deployment of the automated system to data acquired with different acquisition protocols (43).

## Limitations

The major limitation of this study is that it is a retrospective study with a small sample size. However, in light of this proof of feasibility, a larger study is now warranted. The myocardial blood flow values were not compared to a gold standard test or a control group, however, this is a frequently encountered limitation in studies involving pediatrics due to the ethical considerations of this vulnerable age group and the need to avoid unnecessary procedures. Furthermore, coronary anatomical assessment was primarily done via CMRA and not CT or X-ray angiography, despite the latter being the gold standard procedure. However, this is justifiable in the light of the incorporation of CMR as an acceptable modality for coronary assessment in many published guidelines, as well as the reduced radiation exposure.

## Conclusion

Voxel-wise quantification of MBF in pediatric patients is feasible and correlates with the anatomical status of corresponding coronary perfusion vascular territory. This makes use of highly accelerated pulse sequences to achieve sufficient spatial and temporal resolution. Advanced motion compensation and automated image processing methods provide a fully objective interpretation of perfusion, circumventing the need for time-consuming processing and experienced operators. While further studies are need, quantitative perfusion CMR represents a promising tool for the non-invasive management of children with heart diseases.

## Data Availability Statement

The raw data supporting the conclusions of this article will be made available upon request by the authors, without undue reservation.

## Ethics Statement

The studies involving human participants were reviewed and approved by Evelina London Children Local Ethics Committee (Ethics No. RJ109/N112). Written informed consent to participate in this study was provided by the participant's legal guardian/next of kin.

## Author Contributions

CS, HH, PD, and AC designed the study, collected and analyzed the data, and drafted the initial manuscript. GG and TH collected and analyzed the data. RR, JL, and KP designed the study, reviewed, and edited the manuscript. All authors contributed to the article and approved the submitted version.

## Conflict of Interest

The authors declare that the research was conducted in the absence of any commercial or financial relationships that could be construed as a potential conflict of interest.

## Publisher's Note

All claims expressed in this article are solely those of the authors and do not necessarily represent those of their affiliated organizations, or those of the publisher, the editors and the reviewers. Any product that may be evaluated in this article, or claim that may be made by its manufacturer, is not guaranteed or endorsed by the publisher.
